# Hedgehog Signaling Regulates Taste Organs and Oral Sensation: Distinctive Roles in the Epithelium, Stroma, and Innervation

**DOI:** 10.3390/ijms20061341

**Published:** 2019-03-16

**Authors:** Charlotte M. Mistretta, Archana Kumari

**Affiliations:** Department of Biologic and Materials Sciences, University of Michigan School of Dentistry, Ann Arbor, MI 48109, USA; archanak@umich.edu

**Keywords:** chorda tympani nerve, glossopharyngeal nerve, fungiform papilla, circumvallate papilla, geniculate ganglion, trigeminal ganglion, basal lamina, taste bud, sonidegib, hedgehog pathway inhibition

## Abstract

The Hedgehog (Hh) pathway has regulatory roles in maintaining and restoring lingual taste organs, the papillae and taste buds, and taste sensation. Taste buds and taste nerve responses are eliminated if Hh signaling is genetically suppressed or pharmacologically inhibited, but regeneration can occur if signaling is reactivated within the lingual epithelium. Whereas Hh pathway disruption alters taste sensation, tactile and cold responses remain intact, indicating that Hh signaling is modality-specific in regulation of tongue sensation. However, although Hh regulation is essential in taste, the basic biology of pathway controls is not fully understood. With recent demonstrations that sonic hedgehog (Shh) is within both taste buds and the innervating ganglion neurons/nerve fibers, it is compelling to consider Hh signaling throughout the tongue and taste organ cell and tissue compartments. Distinctive signaling centers and niches are reviewed in taste papilla epithelium, taste buds, basal lamina, fibroblasts and lamellipodia, lingual nerves, and sensory ganglia. Several new roles for the innervation in lingual Hh signaling are proposed. Hh signaling within the lingual epithelium and an intact innervation each is necessary, but only together are sufficient to sustain and restore taste buds. Importantly, patients who use Hh pathway inhibiting drugs confront an altered chemosensory world with loss of taste buds and taste responses, intact lingual touch and cold sensation, and taste recovery after drug discontinuation.

## 1. Introduction

**Figure 1:**
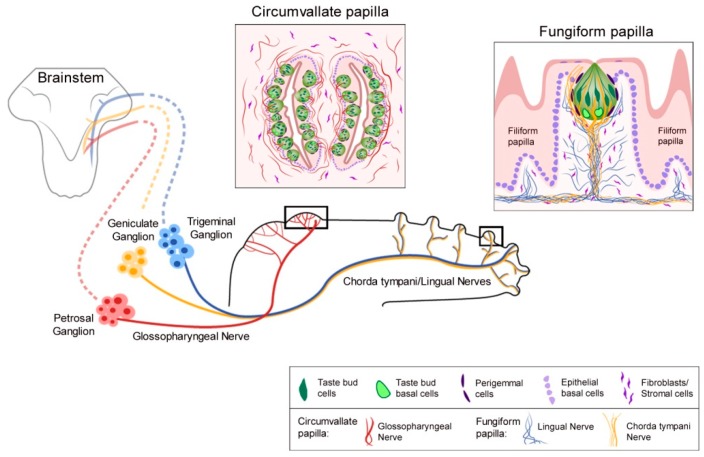
Lingual taste system. The tongue in sagittal section and sensory ganglia are diagrammed, with nerve projections to the anterior and posterior tongue and papillae. Ordered central projections from ganglia to the brainstem are diagrammed in hatched lines. The chorda tympani/lingual nerve projections, from the geniculate (chorda tympani) and trigeminal (lingual) ganglion neurons to the anterior tongue, enter the tongue in a common bundle but redistribute within the papillae to gustatory (chorda tympani) and non-gustatory (lingual) papilla tissues. Glossopharyngeal nerve projections from petrosal ganglion neurons are to the posterior tongue and circumvallate papilla. Not illustrated, the glossopharyngeal nerve also innervates the gustatory foliate papillae in the posterior lateral walls of the tongue. Boxed diagrams are as follows: The circumvallate papilla with multiple and contiguous taste buds in the papilla epithelial walls. The fungiform papilla with a single apical taste bud in the epithelium, covering a broad connective tissue core with stromal cells and innervation. In addition to directly entering into the taste bud, the chorda tympani nerve extends fibers into the apical epithelium. Non-taste filiform papillae bracket the fungiform papilla. The glossopharyngeal nerve innervates taste buds in the circumvallate papilla and papilla connective tissues. The boxed legend refers to elements in the circumvallate and fungiform papilla diagrams.

Sonic hedgehog (Shh) is a principal and essential regulatory molecule in taste bud (TB) homeostasis and taste sensation, demonstrated with multiple approaches, including: When Shh signaling is activated [1,2,3], inhibited [3,4,5,6,7,8], suppressed [9], or if key components are genetically deleted [3,4,6,9], then, in turn, the TBs are altered and taste nerve responses to chemical stimuli are eliminated [5,6,7]. Although TBs are lost and taste nerve responses to lingual chemical stimuli are abolished, notably the nerves are still functional after Hedgehog (Hh) signaling disruption and respond to lingual cold and tactile stroking stimuli [5,6,7]. Consonant with the signaling effects, in the lingual epithelium the Shh ligand is localized within TBs to signal, via paracrine mechanisms, to Hh-responding cells that include the perigemmal cells surrounding the TB, the basal cells of the papilla epithelium, and stromal cells in the taste papilla connective tissue [1]. Further, the ligand is within chorda tympani and glossopharyngeal nerve fibers that distribute in the fungiform (FP) and circumvallate (CV) papilla connective tissue cores, respectively [3,4,6]. Basic lingual and papilla distributions of tongue innervation from the sensory ganglia are diagrammed in Figure 1. The Shh ligand within both TB cells and innervating taste fibers is in a close association with the gustatory papilla basal lamina, a network of molecules that can potentially sequester the Shh ligand to enable numerous functional signaling roles [10].

Therefore, homeostasis of the peripheral gustatory system, that is, the taste organs and gustatory sensation, includes epithelial and connective tissue cell biology and sensory cell neural function. There are recent reviews of Hh signaling and taste biology [11,12,13,14]. Different perspectives are presented here that focus on how Hh signaling regulates cell components of the gustatory epithelium, the stroma, the taste nerves, and nerve–taste papilla cell interactions. Further, there is emphasis on taste and somatosensation transmitted from taste organs. Recent studies demonstrate Shh ligand not only in TB cells but also in innervating fibers, to discern particular roles for the Hh pathway in specialized compartments of epithelium, connective tissue, and nerves [3,4,5,6,9]. These illustrate interest in the current literature. Major sections of this review include a brief discussion of innervation and Hh signaling in taste papilla formation; identification of Shh signaling locations in postnatal and adult tongues; discussion of the essential nature of epithelial Hh signaling in taste organ homeostasis and recovery after pathway disruption; lingual innervation and Hh signaling; distinct sources of Shh ligand and roles in taste homeostasis; taste organ niches and the basal lamina; disrupted Hh signaling and altered oral sensation, in animal and clinical studies; and conclusions and future directions. We have emphasized studies of Hh pathway regulation in FP and TB homeostasis, but also refer to the CV and TBs to illustrate similarities and differences in the posterior tongue effects.

## 2. Development of the Taste Papilla Organ, the Role of Innervation, and Hh Signaling

Before immature or incipient TB cells appear in the tongue epithelium, the lingual innervation grows into the embryonic tongue directed to the developing taste papilla placodes [15]. Thus, the nerves do not grow into the tongue epithelium in a broad distribution and then redirect into constrained papilla placode locations. A working hypothesis of initial exuberant overgrowth into the lingual epithelium, therefore, was not supported. On the other hand, there was no evidence for nerves providing molecules to initiate development of the papilla placodes; rather, the placodes had already begun to differentiate before or concurrent with nerve growth to FPs.

To directly test whether nerves are required for gustatory papilla formation, a whole tongue organ culture system was established that excluded intact innervation and demonstrated that taste papilla placodes form and differentiate in a patterned array without sensory nerves [16]. The hypothesis that taste nerves are essential for gustatory papilla formation, therefore, was not supported. If nerves are not required for taste papilla formation, what factors are crucial? The lingual organ culture system was distinguished by encompassing the entire oral and pharyngeal tongue and became widely used in studies of taste papilla development [17]. It was possible to study regulatory molecules and factors that could direct emergence of the FP in the stereotypical pattern on the anterior tongue, and with avoidance of the broad intermolar eminence [16]. By growing tongue organ cultures from E11 though E18, without an intact sensory innervation, and studying embryonic tongues in vivo, the following was demonstrated:

- The tongue itself requires Hh signaling for initial formation and growth [18].
- In concert with papilla formation and differentiation, Shh location in the lingual epithelium progresses from a homogeneous distribution to placode-specific to confined in the apical papilla [19,20].
- The distinctive FP spatial papilla pattern requires Hh signaling; if the Hh pathway is inhibited, the number of FP double on the anterior tongue and appear, atypically, on the intermolar eminence [18,21].
- There are specific time-dependent effects for Hh roles in tongue formation and growth, FP patterning, and differentiation [20,22].
- Effects of Hh signal disruption on FP and CV papilla development are different [18].

The initial, directed innervation to taste papilla placodes in the embryonic tongue could rely on placodal expression of specific cell and/or molecular attractants for growing nerves without a TB presence, per se. Therefore, it was compelling to determine what molecular signatures characterized papilla and non-papilla tongue regions. With further use of the whole tongue cultures, roles for Bone Morphogenetic Protein (BMP) 2, 4 and 7 [23], noggin and follistatin [23], Epidermal growth factor receptor (EGF) [24], Wnt5a [25,26], Wnt10b [27,28], Fibroblast growth factor (FGF) [29], and retinoic acid [22] were reported in FP formation and differentiation. However, nerve guidance and trophic issues were not directly addressed, although Brain-Derived Neurotrophic Factor (BDNF) has been demonstrated as a chemoattractant that is required for the taste nerve to innervate FP [30,31]. Interestingly, deleting BDNF at E14.5 did not alter taste placode number or Shh expression [32], supporting the data that taste placode formation does not require sensory nerves [16]. Prominently, throughout these developmental studies, there emerged and continued an emphasis on Hh signaling as a major morphogen pathway in the taste system.

## 3. Shh and Shh Signaling Locations Postnatally and in the Adult Tongue and Taste Organs

Given the striking demonstrations of Shh as a major prenatal morphogen that signals in taste papilla formation, differentiation and patterning, investigations extended to postnatal and adult taste system regulation. Postnatal ligand and responding/target cell expressions were identified [1,32,33,34]. Crucial evidence emerged that Shh in the lingual epithelium is only within TB and also that Hh-responding target cells are in TB perigemmal cells, FP basal epithelial cells, and connective tissue cells of the FP stromal core [1]. Paracrine pathways were proposed for the long-range, and diffusible Shh effects in regulating the Hh pathway in the anterior tongue.

Further, with genetic-inducible fate mapping approaches (*Gli1^CreERT2^;R26R^lacZ^*) it was demonstrated that Hh-responding *Gli1^lacZ^*+ basal cells of the FP epithelial trough contribute to cells of the TB, the TB perigemmal cells, apical FP basal cells, and the FP and lingual epithelial cells [1]. Therefore, responding cells in the Hh pathway are progenitors for TB cells. In addition, *Gli1^lacZ^+* cells expressed K5, extending and replicating a prior conclusion that K14+/K5+ cells were contributors to TB *and* lingual epithelial cells [35]. Lineage tracing of *Shh*+ cells (*Shh^CreERT2^;R26R^lacZ^*^/EGFP^*)* suggested that TB basal cells positive for *Shh* are immediate precursors of all TB cell types [36]. Notably, with a transgenic mouse model to activate Hh signaling in K5+ cells (*K*5*^rtTA^;TRE-GLI2ΔN*), TB were lost and FP morphology was disrupted [1]. On the other hand, when the Shh ligand was misexpressed in K14+ basal cells (*K14^CreER^;SHH^YFPcKI^*), ectopic TB or clusters of K8+ cells formed in the non-taste lingual epithelium [2].

Thus, the following was understood: Hh signaling elements in the taste organ epithelium were positioned to regulate taste papilla biology in a paracrine fashion; Hh-responding cells were progenitors for TB, taste organ associated cells, and lingual epithelium; and, disrupting Hh signaling altered FP and TB integrity. With direct identification of Hh signaling components within TB and taste papillae and reports of Hh signaling in epithelial cells as a regulator for TB and taste organ integrity, hypotheses were generated to test how Hh signaling regulated TB homeostasis in the lingual epithelium and to study signaling elements beyond the epithelium.

## 4. Epithelial Hh Signaling is Essential for Homeostasis and Reconstitution of TB and Chemosensation; Innervation Alone is not Sufficient for TB Maintenance

**Figure 2:**
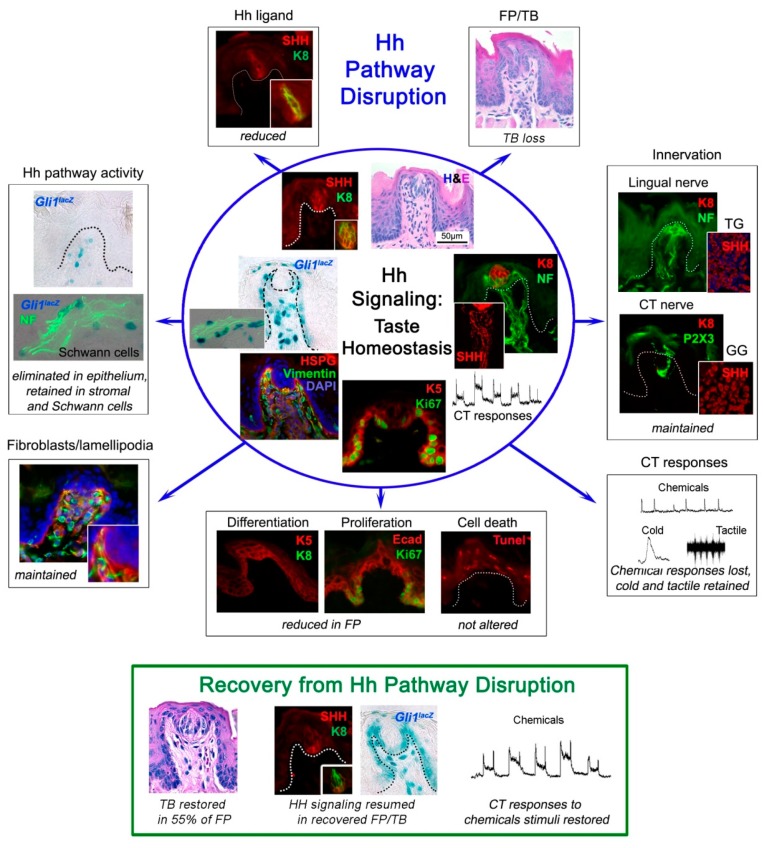
Summary of effects as follows: Hh Signaling Regulation in Taste Homeostasis; Hh Pathway Disruption; and, Recovery from Hh Pathway Disruption. Blue Circle, Inside: Hh signaling regulation in maintaining taste organs and sensation. Hh Signaling in Taste Homeostasis is illustrated in the fungiform papilla taste organ (FP), taste bud (TB), and Shh ligand (top); papilla and taste bud innervation and chorda tympani (CT) neurophysiological responses (right); taste organ cell functions (bottom); connective tissue fibroblasts (bottom left); and Hh pathway activity in papilla epithelium and stroma (left). Blue Circle, Outside: Effects of Hh Pathway Disruption by genetic suppression or pharmacologic inhibition. With Hh Pathway Disruption, blue arrows followed from the top and proceeding clockwise, there is the following: taste bud loss and associated reduction of Shh ligand (top); maintained innervation and ganglion neurons; elimination of taste responses but retained responses to cold and touch; reduced cell proliferation, compromised differentiation with unaltered cell death; sustained fibroblast/lamellipodia activity; and, loss of Hh signaling in the papilla epithelium, but retention in the connective tissue core. Green Box: Recovery from Hh Pathway Disruption. The following occurs after pathway disruption is discontinued: TB are restored in about 55% of FP; Hh signaling is resumed in epithelium of recovered FP; and, taste responses are restored. (Results in the figure are drawn principally from hematoxylin and eosin staining, immunohistochemistry, X-gal staining, and electrophysiology. Effects are illustrated with the FP/TB but apply also to the circumvallate papilla and taste buds). Some of the images for Taste homeostasis (inside Blue Circle), Hh pathway disruption (outside of the Blue Circle), and the entire Recovery panel (Green Box) are extracted from Figures in Kumari et al. 2017 [6] with permission. Scale bar in H&E image within the blue circle applies throughout.4.2. Hh Signaling Disruption with Genetic Models.

To learn whether the Hh pathway could regulate the constantly renewing TB cells in adult rodents and thereby support homeostasis in a sensory organ system with receptor cells that continually turnover [37], Hh signaling was altered pharmacologically and genetically in the whole body or in epithelial tissues [5,6,7,9]. Furthermore, the potential for taste organ recovery was investigated after discontinuing Hh pathway disruption. Overall, a direct requirement for Hh signaling within the lingual epithelium was shown for TB functional homeostasis and reconstitution in recovery, summarized in Figure 2.

### 4.1. Hh Pathway Inhibition with Sonidegib: Taste Organ and Sensory Functional Effects

Kumari et al. 2015 [5] studied the Hh pathway in taste sensation, using the cancer drug sonidegib (LDE225) to pharmacologically inhibit signaling at Smoothened, a major upstream regulator of Hh signaling. Once the Hh ligand binds to the membrane receptor Patched, Smoothened is released from Patched-inhibition and initiates a signaling cascade that transmits signals to activate Gli transcription factors [38]. With sonidegib treatment, a direct and essential requirement of the Hh pathway in TB maintenance and functional homeostasis was demonstrated [5] (Figure 2). The focus was on TB of the anterior tongue FP and the study was groundbreaking in addressing Hh signaling in neurophysiological taste sensation. TB rapidly and completely deteriorated so that by 16 days there essentially were no intact TB in FP.

Markedly, however, general lingual nerve (LN) and taste-specific chorda tympani (CT) innervations into the FP core (Figure 1) were maintained. In recordings from the CT nerve there was a loss of responses to chemical stimulation of the tongue, predicted from the TB elimination (Figure 2). Notably, it was discovered that Hh signaling has different roles in chemosensory and somatosensory receptor and neural response maintenance because CT nerve responses to lingual cold and mechanical stroking stimuli were not eliminated (Figure 2; and see later Discussion, Section 8).

Extending studies from Hh Pathway Inhibition (HPI) with the cancer drug sonidegib, effects on TB in the CV as well as the FP were investigated and the validity of prior HPI conclusions was affirmed [6,7,9]. In genetic epithelial approaches, K5-targeted mouse models were used for (1) *Hh/Gli* suppression (*K5^rtTA^*;*tetO-Gli2ΔC4* and *K5^Cre^*;*R26^LSLrtTA^*;*tetO-Gli2ΔC4)*; (2) deletion of the *Gli2* transcription factor (*K5^rtTA^*;*tetO-Cre*;*Gli2^fl/fl^* and *K5^rtTA^*;*tetO-Cre*;*Gli2^fl/fl^Gli1^lacZ/lacZ^*); and (3) *Smo* deletion (*K5^rtTA^*;*tetO-Cre*;*Smofl/fl*). In these models, the time course and extent of effects on FP and CV TB were comparable to pharmacological Hh pathway blockade [6,7] and to global *Smo* deletion (*R26M2rtTA/+*;*tetO-Cre*;*Smofl/fl*) [6], emphasizing the crucial role of epithelial Hh signaling in TB homeostasis.

Overall, in combination with studies using the HPI drug sonidegib, it was demonstrated that with Hh signaling disruption the TBs were rapidly lost and Shh ligand within the TB was reduced with the loss of TB cells (summarized in Figure 2, illustrated with FP and TB). This was attributed both to reduced proliferation in basal cells of the FP and to a loss of apical epithelial differentiation potential; cell death was not significantly affected [6,9]. Vimentin+ stromal cells and lamellipodia were not obviously altered, and the FP connective tissue core retained *Gli1^lacZ^*+ Hh-responding cells, in epithelial and whole-body genetic models [9]. Further, innervation to the taste organs was retained and the fibers were closely associated with the *Gli1^lacZ^*+ Hh-responding Schwann cells in the stroma [9]. Thus, there were major effects in the taste organ epithelium and TBs, whereas connective tissue elements and nerves were maintained (Figure 2). Across models, an essential role for, and strict dependence on, epithelial Hh signaling in TB maintenance in FP and CV papillae were shown. Further, the seminal observation was made that nerves alone are not sufficient to sustain TB in the absence of Hh signaling integrity in the epithelium.

In other studies, effects of the Hh pathway on proliferation and differentiation of TB progenitors in the FP, without effects on cell death, were replicated with pharmacologic HPI [3]. Prior work, with epithelial activation of Hh signaling, also demonstrated hyperproliferation and a differentiation defect in the FP apex [1]. With misexpression of Shh in K14+ basal cells, Shh-induced ectopic ‘taste buds’ or clusters of K8+ cells were observed, surrounded by *Gli1^lacZ^+* cells. The K8+ cells lacked innervation and were located throughout non-gustatory papilla regions of the tongue epithelium, suggesting that Hh signaling can direct TB cell type differentiation [2]. In a recent functional study, the *Gli3* transcription factor was located in TB of FP and CV, highly expressed within stem cells (Lgr5+ epithelial cells) and *Tas1r3+* taste receptor cells of the CV [39]. With a conditional deletion of *Gli3* there was an increase in number of TB cells and expression of *Tas1r3+* in taste cells. Therefore, *Gli3* was reported as a negative regulator of differentiation and survival of *Tas1r3+* taste cells, with effects on bitter and sweet taste sensation. These additional studies reinforced roles for intact epithelial Hh signaling in TB homeostasis.

### 4.2. Recovery from Hh Pathway Disruption

To address the potential for recovery after Hh pathway inhibition/suppression, the signaling blockade was removed and animals recovered for a period of a few days to several months [6,7,9]. Intriguingly, the FP and CV had different recovery patterns. Although the FP/TB recovery was dependent on the duration of Hh/Gli suppression, without reconstitution after a very long treatment period, the CV/TB recovery was complete even after a prolonged pharmacological blockade [6,7,9]. The FP/TB completely recovered after 5 days of Hh/Gli suppression [9], whereas extending the suppression to 16 days led to reconstitution of about 55% of the FP/TB after 14 days, or up to 9 months, of recovery (Figure 2) [6,9]. When animals were treated for 48 days with sonidegib, there was no restoration of FP/TB even after discontinuing the drug for 7 months [7]. Overall, restoration effects were comparable after pharmacologic Hh pathway inhibition or after Hh signaling blockade in the epithelium [6,9]. Regeneration was always accompanied by Hh signaling within the epithelium and this occurred when at least some TB cells and associated Shh expression were present.

**Figure 3:**
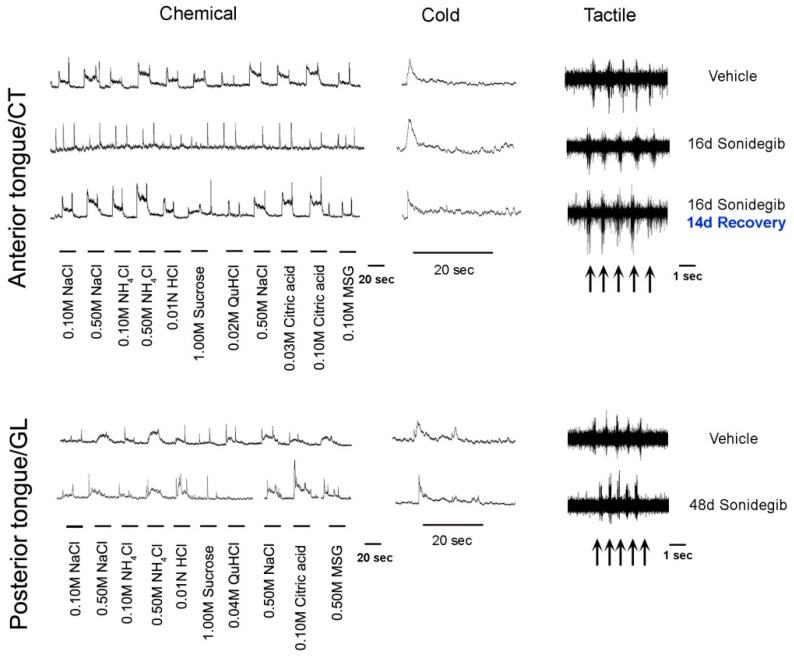
Gustatory nerve responses from innervation to anterior and posterior tongue taste buds. Whole nerve recordings from the chorda tympani nerve (CT) innervating taste buds in the anterior tongue and the glossopharyngeal nerve (GL) innervating taste buds in the circumvallate papilla and surrounding tissue of the posterior tongue. Responses to chemical, cold water (4 °C), and tactile stroking stimuli were recorded in Vehicle- and Sonidegib-treated rodents and for the CT after 14 days recovery from Sonidegib treatment. After Hh pathway inhibition with Sonidegib the responses to chemical stimuli were eliminated (CT) or substantially reduced (GL). However, responses to cold and tactile stimuli were maintained, indicating a modality-specific role for the Hh pathway in lingual sensory function. In recordings from the CT after 14 days recovery from Sonidegib treatment, responses to taste stimuli returned in concert with taste bud recovery [6,7]. Recording images for Anterior tongue/CT are extracted from Figure 8 in Kumari et al. 2017 [6] with permission, and for Posterior tongue/GL are extracted from Figure 6 in Kumari et al. 2018 [7] with permission.

Notably, when TB recovered in 55% of FP after withdrawing Hh signaling suppression, this partial recovery was accompanied by fully restored CT whole nerve chemosensory responses (Figure 2) [6]. Within the CV, even after prolonged treatment, there were retained TBs and moderate glossopharyngeal nerve (GL) nerve responses (Figure 3) [7]. The implications of recovery have been noted in relation to patient-reported taste disturbance with use of Hh pathway inhibiting drugs. A further discussion of recovery from Hh pathway disruption is in Section 6.1., including activation of the pathway during recovery using the Smoothened agonist (SAG) [3].

### 4.3. Nerves are not Sufficient for TB Maintenance or Restoration after Hh Signaling Disruption

After *Hh/Gli/Smo* suppression, robust P2X3-(CT) and NF-(CT/LN) labeled innervation was observed within FP that had substantially reduced or no TB cells (K8+ labeled), compared to control FP with intact TB (Figure 2). There was no general misdirection or loss of innervation in the anterior tongue over extended periods, yet TB were not maintained or rescued from elimination [5,6,9]. Hh-responding *Gli1^lacZ^*+ cells were adjacent to nerve bundles and included S100b-labelled Schwann cells within the FP and along fascicles within the body of tongue [9]. Nerves were also retained within CV papillae after HPI with sonidegib or *Hh/Gli/Smo* deletion, although TB were eliminated, as in the FP [6,7,9]. In pathway inhibition using sonidegib, the geniculate ganglion neurons and their Shh expression were maintained (Figure 2). Overall these data suggested that TB require epithelial Hh because the remaining nerves could not sustain Hh signaling in the epithelium [5,6,9]. Furthermore, when animals were allowed to recover from pharmacological Hh signaling blockade, or *Hh/Gli* suppression, FP and CV that had TB remnants only, and thus some Shh expression, were restored [6,7,9]. Strikingly though, FP without any TB or Shh in the epithelium, that still retained innervation, could not recover even after removing the signaling inhibition for 7–9 months [6,7]. Therefore, Hh signaling within the lingual epithelium was shown to be necessary for TB cell maintenance and restoration. Intact innervation that remained after Hh signaling disruption, although replete with Shh ligand, was not sufficient for TB homeostasis and renewal.

## 5. Lingual Innervation and Hh Signaling: TB Maintenance and Regeneration in Nerve Cut Studies and BDNF Requirement

In view of effects of epithelial Hh signaling suppression and remaining innervation, the complexities of taste bud cell dependence on sensory nerves have been noted [9]. Strikingly, the retained innervation and Hh-responding connective tissue cells of the papilla during epithelial *Hh/Gli/Smo* blockade were not sufficient to maintain TB cells without epithelial Hh signaling integrity. Whereas it was shown that Hh signaling within the lingual epithelium is essential for TB maintenance, the long-documented nerve-dependent degeneration and regeneration of TB had demonstrated that nerves clearly are essential, too, in TB homeostasis [40,41,42].

### 5.1. Nerve-Dependence and Degeneration/Regeneration of Taste Buds

Both chorda tympani (CT; with neuron soma in the geniculate ganglion, GG) and lingual (LN; with neuron soma in the trigeminal ganglion, TG) nerves innervate the anterior tongue and FP (Figure 1). The CT and LN enter the tongue in a common bundle. However, within the taste organ, the CT is directed toward and into the TB, while the LN extends throughout FP core area and densely below the TB without innervating the TB. In the posterior tongue, the CV and TB are innervated by the glossopharyngeal nerve (GL; with neuron soma in the petrosal ganglion) (Figure 1). Since taste bud cells turn over and are constantly renewed [37], a process of continued reinnervation is part of TB receptor cell homeostasis. The inability of nerves in the FP and CV papilla connective tissue cores, and extending to the epithelium, to sustain or renew TB after Hh pathway inhibition or signaling suppression [5,6,7,9] was somewhat surprising given the longstanding tenet that TB depend on lingual innervation.

In addition to ongoing homeostatic renewal, it had been known for decades that TB degenerate after nerves are severed and then regenerate after nerves grow back into the tongue. Guth [43] and Zalewski [44] extended the classic work of von Vintschgau and Honigschmied [45] and documented time courses for degeneration/regeneration of TB in the CV. GL nerve cut led to loss of neural and TB markers and their receptors [46,47,48,49]. In addition, the taste nerve dependence of TB was shown in applications to the FP taste organ [40,50] and distinctions were drawn between roles for LN versus CT nerves in FP TB dependence [42,51,52]. When the CT, either alone or together with the LN nerve, was transected there were extreme alterations in TB number and FP morphology [40,42,52,53,54], loss of taste sensation [55], and reduced expression of neural markers within TB [56].

Although regeneration of taste nerves and TB is long-studied, there had been no one factor identified that supports this regeneration. The neurotrophin BDNF was considered as a potential and essential TB supporting factor because BDNF is necessary for TB innervation during development [31] and is required for innervation in the adult taste system [57]. Importantly, it was demonstrated recently that, after nerve cut, the neurotrophin BDNF is essential for taste nerve regeneration and reinnervation of TB [58]. BDNF is not depleted but remains in the GG and lingual epithelium after the nerve cut. Moreover, BDNF maintains a subset of nerve fibers that innervate TB (about 40% of the total) and these fibers express the full length trkB receptor [59]. Thus, BDNF is firmly established as a required factor for TB maintenance and regeneration.

A potential association between BDNF and Shh signaling has not been studied in the tongue. However, examples for Hh pathway regulation of BDNF expression have been shown in vitro in cortical neurons [60,61], in the spinal cord [62], and in regulation of normal cavernous nerve and nerve regeneration following nerve crush [63]. Additionally, BDNF can induce Shh expression [64,65,66,67]. Focused studies are required; however, interactions are likely between Shh and BDNF for the maintenance and renewal of lingual and taste organ innervation and in BDNF-mediated nerve regeneration.

### 5.2. Innervation and Shh Signaling

Nerve cut studies also reveal that innervation is required for the expression of Hh signaling components in TB. GL nerve transection resulted in rapid loss of Shh within basal cells before degeneration of the TB and there was re-expression of Shh before the formation of TB during nerve regeneration, suggesting a nerve-dependent expression of Shh in TB [68]. CT nerve transection also reduced Shh expression in the anterior tongue epithelium [3]. In studies with a non-regenerative CT/LN nerve cut there was retention of Shh at moderate levels in the lingual epithelial TB remnants and elimination of the Shh in those FP without any TB [69,70]. These data point to involvement of Hh signaling in nerve-dependent degeneration/regeneration of TB.

In another system, the cutaneous nerves in skin carry Shh [71] and innervate Hh-responding cells in the touch dome [72] to activate Hh signaling. With nerve cut there is a reduction of Hh-responding, *Gli^1lacZ^*+ cells and Merkel cells in the touch dome [73]. Thus, to maintain Hh pathway activity, the innervation is required and nerve-derived Shh is suggested as a neural factor in the touch dome niche [73].

### 5.3. Removing the FP Organ and Hh-Responding TB Progenitors

With a different experimental perspective, an intriguing paper for the study of taste organ regeneration demonstrated that if the entire FP is dissected, in comparison with just the apical half of the papilla, TB would not regenerate within the tongue epithelium where the FP had been located [74]. There was a failure to regenerate although the tongue was fully innervated. Removing the entire FP effectively eliminates all proliferation niches for Hh-responding, progenitors of the FP and TB, whereas dissecting only the apical half leaves the progenitor compartment intact at the FP base [1,13]. In addition, the FP clearly has some unique element(s), not included within the general lingual epithelium, for TB homeostasis and regeneration. Notably, in the anterior tongue the TB are located only within FP, typically and after neural regeneration or recovery from HPI, indicating the specific nature of the FP as a residence for TB. In summary, the importance of the gustatory organ epithelium in maintaining taste homeostasis is supported.

## 6. Sources of the Shh Ligand in TB Cells and in Nerves; Potential Distinctive Roles

In the taste organ Hh-responding, FP epithelial basal and perigemmal cells, and connective tissue cells were observed [1,5,6,9]. With epithelial *Hh/Gli* signaling suppression, *Gli1^lacZ^+* cells were eliminated from the FP epithelium but the pathway remained active in the stroma [9] and *Gli1^lacZ^*+ cells were closely associated with nerves (Figure 2). There was a possibility that the TB remnants in Atypical FP/TB, which contain Shh ligand [5,9], could signal to elements in the FP core. However, in Atypical FP with no TB, and therefore no epithelial Shh ligand, *Gli1^lacZ^*+ Hh-responding cells were still observed in the FP connective tissues. This suggested potential ligand sources that maintain pathway activity in the stromal core, in the context of no TB cells or epithelial Hh. Nerve fibers in the tongue had been proposed as a source of Shh [13], which was later shown experimentally by Kumari et al. (2017) [6], Castillo-Azofeifa et al. (2017) [4], and Lu et al. (2018) [3].

With knowledge of at least three sources of Shh ligand observed within the peripheral taste organ system (in TB, GG, and TG), experiments were designed to determine whether and how signaling in the epithelium and/or nerves might function in Hh regulation of the adult taste periphery [3,4,6]. From 2015 it was emphasized that nerves alone were not sufficient to maintain TB if Hh-signaling integrity was compromised in the taste organ epithelium [5]. However, based on direct information that the Hh ligand was within taste nerves, different approaches were applied to consider individual roles for each source, from nerves and epithelium, in Hh pathway activity, and in TB maintenance [3,4].

### 6.1. Sources of the Sonic Hedgehog Ligand and Signaling in TB Cells and in Ganglion Soma and Nerves

To distinguish among roles for the Hh ligand in TB or in nerves, various mouse models were used [3,4,6] including:*Shh* reporters to localize *Shh* (*Shh^CreER^*;*R26^RFP^ and Shh_CreERT2_*;*R26R^tdTomato^* and *Shh^CreER/^*^+^;*R26^mTmG^*); *Shh* deletion from epithelium (*K5*;*ShhcKO*:*Krt5^rtTA^;tetO^Cre^*;*Shh^flox/flox^*) or nerves (*Advillin*;*ShhKO*:*Avil^Cre/+^*;*Shh^flox/flox^* and *Thy1*;*ShhcKO*:*Thy1^CreER/+^*;*Shh^flox/flox^* and *AAV5*;*ShhcKO*:*AAV5^Cre^*; *Shh_CreERT2/flox_*;*R26R^tdTomato^* and *Shh;ShhcKO*:*Shh^CreERT2/flox^*;*R26R^tdTomato^*); and, Hh signaling antagonists (including sonidegib; vismodegib; XL139; HhAntag). Shh ligand expression was confirmed within TB cells and reported in GG and TG soma [3,4,6] and CT and GL nerves [3,4,6]. Shh localization within LN nerves fibers in the taste organs was not addressed.

When *Shh* was deleted specifically from epithelial cells (*K5*;*ShhcKO*) [3,4], Shh expression was reduced in TB but there was no change in TB number (K8+ labelled cells). It was suggested that the loss of epithelial Shh alone did not alter TB maintenance. Based on numbers of Typical TB remaining and retained *Gli1* expression in the lingual epithelium, after *Shh* deletion from the epithelium, it was proposed that neural Shh can support TB renewal and epithelial Hh signaling [4]. However, with a focus on Hh signaling suppression in the epithelium, there was a significant reduction in Typical TB and Shh ligand, and elimination of *Gli1^lacZ^*, Hh-responding cells from the papilla epithelium (Figure 2) [6,9]. Overall, Shh from any TB remnants *or* retained innervation was apparently unable to signal to the epithelium when *Gli2/Smo* was suppressed or deleted from K5+ cells.

To delete Shh specifically from nerves innervating the FP, various models were used by Castillo-Azofeifa et al. (2017) (*Shh*;*ShhcKO*; *AAV5*;*ShhcKO*) [4] and Lu et al. (2018) (*Advillin*;*ShhKO*; *Thy1*;*ShhcKO*) [3]. Innervation density in typical FP and the number of TB remained unaltered after removing neural/GG *Shh* for 5 weeks and ‘redundant’ roles for neural and epithelial sources of Shh were proposed [4]. When a neural deletion was extended to 12 weeks using a *Thy1CreER* mouse, the numbers of TB containing K8+ cells were somewhat reduced, by about 25% [3]. However, with a constitutive neuronal Shh knockout (*Advillin*;*ShhKO*), about 85% of TB that contained K8+ cells were lost by 20 weeks postnatal. There were no differences in this model at 8 weeks, although it was a constitutive deletion [3]. It was concluded that neural Shh contributed to long term TB maintenance and regeneration. In skin, using the same *Advillin*;*ShhKO* model, it had been shown that neural Shh is necessary for long term maintenance of the touch dome and K8+ Merkel cells [72].

Extended deletion of Shh from nerves included ablation from all neuron cell bodies [3]. Of the two nerves, CT and LN, that innervate the anterior tongue (Figure 1), Shh expression has been demonstrated in the CT nerve and cell soma of the GG (Figure 2) [3,4,6]. These are the direct innervation to sustain TB [42]. The TG neurons also have Shh+ cells [4,6]. Although it has not yet been shown if the LN nerve transports Shh from TG cell soma to the FP, it was reported that the inferior alveolar nerve fibers carry Shh from TG to the mouse incisor mesenchyme [75]. If LN fibers within the taste organ are Shh+ we suggest that they are involved in FP maintenance, not in TB homeostasis per se.

To block signaling from all Shh sources pharmacologic Smo antagonists for HPI were used (sonidegib; vismodegib; XL 139; and HhAntag) and there was a reported minor decrease in TB size in CV after prolonged vismodegib treatment [8], a robust loss of TB cells in FP [3,4], or a rapid elimination of all TB, including those in the soft palate [5,6,7]. Shh+ innervation and numbers of GG neurons were maintained [3,6]. Within the FP and TB there was reduced proliferation and differentiation among Hh-responding cells or TB progenitors and cell death was not a major contributor to Hh signaling effects [3,6] (Figure 2). These FP/TB effects effectively replicated earlier conclusions with epithelial *Hh/Gli* suppression [9].

Using a genetic model to delete Shh from TB cells and innervation concurrently (*K5-AAV-ShhcKO* mice), TBs were essentially eliminated [4]. The investigators concluded that TB maintenance required epithelial and nerve-derived Shh in concert, confirming effects of whole body Shh signaling suppression [6,9] and effects of treatment with the Hh pathway inhibitor drug sonidegib [5,6].

In studies of recovery from Hh pathway inhibition with pharmacologic antagonists (sonidegib, Kumari et al. (2017) [6]; XL 139, Lu et al. (2018) [3]) the restoration of TB was incomplete, even after periods when the drug had been withdrawn for 7–9 months [6,7] and signaling recovery was documented within the taste organs [6] (Figure 2). In FP with restored TB or TB cell remnants there was associated Shh ligand and Hh signaling activity within the epithelium, whereas there was no ligand or Hh signaling in the FP epithelium with no TB [9]. The LN and CT nerves were maintained throughout the periods of pathway inhibition and recovery after drug withdrawal. Additionally, during treatment, Shh expression in the GG was not reduced and GG neurons were not lost [6].

Although intact innervation could not lead to complete recovery/restoration of TB in the absence of epithelial Hh [6], neural Shh was reportedly required for the partial regeneration [3]. Notably, the extent of regeneration of TB (K8+ labeled cells) was a function of Shh gene dosage [3]. Furthermore, the ‘non-recovered’ FP were tractable to recovery because in gavage with a Shh agonist, SAG, there was a 1.9% fold increase in numbers of TB with K8+ cells [3]. However, a principal effect of SAG treatment was to stimulate expression of ectopic K8+ cells in the extra-papilla epithelium throughout the anterior tongue. These cells were not innervated [3]. This echoed results from misexpression of Shh in K14+ cells that led to ectopic K8+ cells, outside of FP, without innervation [2]. Overall, Shh expression could not attract innervation to K8+ cells.

Conclusions from these recent papers have been varied and include the following: (a) TB and nerve sources of Shh are necessary to maintain FP/TB because Shh loss from only one source has minimal effect on TB maintenance and the epithelial and neural sources of Shh function ‘redundantly’ [4]; (b) epithelial Shh deletion does not result in TB loss [3,4], whereas neural Shh contributes to long term maintenance of TB and is required for regeneration of TB [3]; (c) *Hh signaling* in the lingual epithelium is essential for TB morphologic and sensory homeostasis and regeneration [6,9]; and (d) even sustained neural Hh signaling alone, from intact GG and CT fibers, cannot maintain homeostasis or initiate reconstitution/regeneration of TB in the face of epithelial Hh pathway suppression [6,9]. Overall, it is apparent that the FP and the CT each has particular attributes for TB homeostasis and regeneration. We propose that Hh signaling in each is necessary for TB homeostasis, but neither alone is sufficient (see Discussion 6.2, 9.1).

### 6.2. Distinctive Roles for Hh Signaling in the Epithelium Versus Stroma

**Figure 4:**
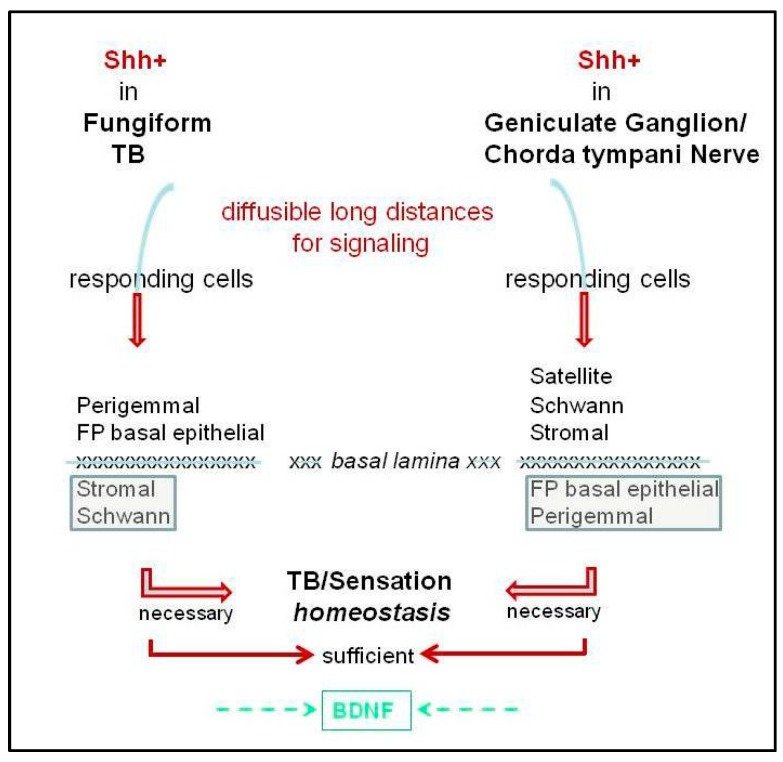
Proposed Hh signaling from Shh ligand in fungiform papilla taste bud and in innervation. Each is necessary for taste bud homeostasis, but only signaling from both is sufficient. The Shh ligand is within taste bud cells (TB) and geniculate ganglion neurons/chorda tympani nerve fibers. The ligand can signal over long distances to responding cells in different compartments of the fungiform papilla and ganglia and tongue. Paracrine signaling from Shh in TB cells has been demonstrated [1,3,13] and signaling from Shh in ganglion/nerve fibers is proposed [3,4,6,13]. If Shh is sequestered within or can traverse the basal lamina, then signaling from both sources will potentially cross to compartments beyond the papilla epithelium (left) or nerve/stromal tissues (right). We propose that signaling in the epithelium and via innervation each is necessary for TB homeostasis and sensation, but only together are they sufficient. BDNF is added at the bottom of the diagram to acknowledge the roles of this neurotrophin in TB homeostasis [57,58,59].

With two broadly designated sources of Shh in the taste organ, in TB and in nerves, the question arises whether each source of Shh ligand signals to both FP epithelium and stroma or specifically to one tissue location (Figure 4). In the skin, neural Shh from the dorsal root ganglia maintains epithelial touch domes [72,73]. In FP, nerves penetrate the basal lamina to enter the TB, which could bring an additional Shh source into TBs and, with paracrine processes, effect signaling to the perigemmal cells or FP epithelial cells (Figure 4). Furthermore, there are Shh+ fibers surrounding the TB and reaching into the apical epithelium [6], which could signal to the *Gli1^lacZ^*+ Hh-responding cells at the FP apex [13].

Additionally, HPI data suggest that Shh+ nerves within the FP core also signal to the FP connective tissue elements (Figure 4). In HPI studies, or with *Gli2* or *Smo* deletion from K5+ basal cells, there was loss of *Gli1_lacZ_* expression from the perigemmal and basal cells of the epithelium in the three s designated types of FP and TB: Typical FP/TB, Atypical FP/TB and Atypical FP/No TB. However, Hh-responding cells remained in the stromal core of all three FP types [6,9]. Since Atypical FP that lack TB, and thereby lack epithelial Shh, still retain stromal *Gli1_lacZ_+* Hh-responding cells, it had been proposed that Shh ligand from the innervation is maintaining Hh signaling activity in the Hh-responding cells of the FP stroma [6].

Similarly, activating a *Gli2* transgene for *Hh/Gli* blockade in K5+ cells resulted in loss of epithelial Hh signaling in all three FP types [9]. However, when mice were allowed to recover from epithelial *Hh/Gli* suppression, about 55% of the altered taste papillae were restored to Typical FP/TB with simultaneous recovery of *Gli1_lacZ_* expression in the FP epithelium. Importantly, in the unrecovered Atypical FP/No TB, that did not have epithelial Shh and no longer had epithelial Hh signaling inhibition, the Hh-responding *Gli1^lacZ^*+ cells were detected only in the FP stroma and not in the basal or perigemmal cells of FP [9]. The data suggest that perhaps a neural Shh source in the FP core signals principally to the papilla stroma and not to the FP epithelium. This further emphasizes that, although the innervation is retained in these Atypical FP without any TB, nerves alone and Shh in nerves alone cannot maintain TB. The same pattern of *Gli1^lacZ^* expression was observed during the recovery from HPI with sonidegib treatment [6]. There were no *Gli1^lacZ^*+ Hh-responding cells in the epithelium of Atypical FP/No TB, even after 21 days of drug removal. The data indicate that there would be no epithelial signaling without the presence of the Shh within the TB and that the nerve source within the FP connective tissue core does not signal to FP basal epithelial cells to regulate proliferation and differentiation. Therefore, this suggests that Hh signaling activity in the FP stromal core is maintained principally by the Shh+ nerve fibers in the FP stroma and that this neural signaling can also maintain Hh-responding Schwann cells and, therefore, neural integrity. This is similar to adult mouse incisor, where Shh from nerve bundles prinicipally maintains stromal, mesenchymal stem cells [75].

However, it is possible that the Shh source from the TB can be sequestered by or cross the basal lamina to activate Hh signaling in the FP stromal and Schwann cells (Figure 4). More studies are required to determine if the TB source of Shh signals exclusively to FP epithelium and not to the FP stroma. Although the collective data suggest that the two sources of Shh have some independent functions, both sources are required to maintain overall FP/TB homeostasis.

## 7. Taste Organ Niches and the Basal Lamina in Hh Signaling

Studies of the Shh ligand and signaling locations in the tongue epithelium and innervation suggest functions for various niches within the taste papilla organs and the surrounding lingual tissues [1,13]. As a highly specialized molecular, cell, and tissue environment, the niche supports stem and progenitor cell functions [76,77,78,79]. Thus, niches are essential in adult taste organ homeostasis, recovery, and regeneration. Albeit not widely studied, there are, strikingly, several niches that are salient to taste organ biology [13].

**Figure 5:**
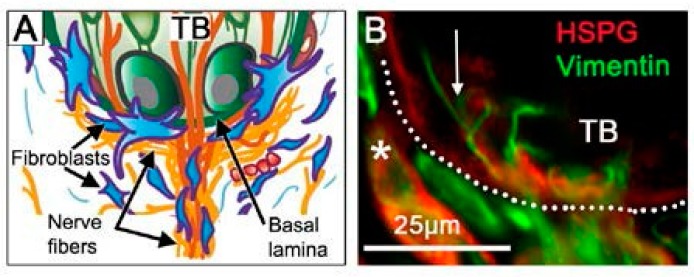
Basal lamina niche region under the taste bud and at the apex of the fungiform papilla connective tissue core. (**A**) Diagram for structural interactions among nerve fibers, fibroblasts and lamellipodia, and taste bud (TB) cells, at the basal lamina (Adapted from Figure 4 in Mistretta and Kumari (2017) [13] with permission) (**B**) Photomicrograph of basal lamina (HSPG immunoreaction, red) under TB cells with lamellipodia extensions (arrow) across the basal lamina from fibroblasts (Vimentin immunoreaction, green). The dotted line demarcates the basal epithelium. The asterisk denotes labelling of blood vessel basal lamina. We propose that Shh is sequestered at the basal lamina in this niche region.

The basal lamina (basement membrane) in the FP apex is centrally positioned in a defined niche region for TB homeostasis (Figure 5) [13]. To traverse or navigate through the basal lamina in physiologic processes, several molecular and cell strategies are used, for example, invadopodia extensions, accessing perforations, and proteolysis [80]. Further, basal lamina components can alter cell fate [81]. The basal lamina incorporates a network of molecules, including heparan sulfate proteoglycan, which is known to sequester the Shh ligand [82]. Seated just under the TB cells that are a rich source of Shh, and at a nexus of nerves coming into the TB that are another source of Shh, the basal lamina might function to sequester the Shh ligand at high concentration and thereby generate gradients for signaling in the epithelium and stroma. The lamellipodia of Shh-responding fibroblasts extending into the basal lamina could access ligand (Figure 5) and the Hh-responding Schwann cells, around the nerves that penetrate the basal lamina to innervate TB cells, might also access ligand in this niche.

## 8. Chemosensation, Somatosensation, Hh Signaling, and Disrupted Taste Perception

Intact oral sensation is critical in making food choices and essential in flavor perception [83]. Sensory information generated from TB receptor cells is transmitted by the gustatory innervation to the central nervous system. Thus, any disruption of either the TB or the innervation, both of which are Shh sources, or deregulated Hh signaling can disturb oral sensation.

In patient-reported taste effects, with the Hh pathway inhibition (HPI) cancer drugs sonidegib and vismodegib [84,85,86], there is scant consideration of overall flavor perception. However, reports of ‘food tastes bad’ and ‘I cannot taste’ [84] might well include flavor components of taste, olfaction, temperature, and touch. The relevance for patients of the modality-specific HPI effects on CT nerve responses have been noted from 2015 and continuing [5,6,7,13]. Neurophysiological taste responses are lost (CT) or much reduced (GL) after Hh pathway inhibitory drug treatment while there are remaining responses to touch and cold in the CT and GL nerve (Figure 3) [5,6,7]. Therefore, roles for Hh signaling in the peripheral taste system are modality-specific. In summary the oral sensory world experienced after HPI, with touch and temperature sensation but no taste, is radically different from intact lingual sensation.

### 8.1. Lingual Taste, Touch, and Cold Sensation after TB Alterations by HPI Drugs: Animal Studies

New knowledge about Hh signaling regulation of taste organs and sensation has derived from neurophysiological responses from two major taste nerves, the CT and GL, during HPI (Figure 3) [7]. Through these recordings it became apparent that with TB elimination there was concomitant loss of responses to chemical stimuli on the tongue [5,6,7]. However, responses to stroking and cold stimuli remained. Therefore, there are modality-specific effects of HPI and these somatosensory responses from the CT and GL did not emanate from TB [5,6,7]. Although TBs, modified epithelial structures, are lost under HPI, the number of GG ganglion soma are not affected [3,6]. Therefore, it is these soma and fibers that innervate the somatosensory receptor cells/organs that continue to respond in the CT during HPI. The GDNF-Ret signaling pathway has been shown to have direct involvement in mediating the somatosensory responses from the CT [87]. Notably, CT fibers respond to somatosensory stimuli, as does the trigeminal ganglion innervation.

Furthermore, it is apparent that the innervation remaining during HPI, even over long periods, cannot sustain or ‘regenerate’ TB in this epithelium [6]. An epithelium that has intact Hh signaling is required for TB homeostasis and renewal. Overall these new findings suggest probing for deeper knowledge about Hh signaling regulation of TB/nerve/ganglion cell and lingual sensory function.

Very recently a species generalization from mouse to a rat has been shown for the main taste effects of HPI [7]. The results from the rat have similarities to, and differences from, mouse data. In general, effects in rat FP and CV and neural responses are more profound than those in mouse. The generalization of HPI effects to another rodent is important and suggests care in applying data to patient concerns. A more nuanced approach to considering taste dysfunction in patients who use HPI drugs is warranted.

### 8.2. Lingual Taste Sensation after Nerve and Taste Bud Disruptions: Patient/Clinical Studies and Hh Signaling

The CT, LN, or GL nerves that innervate the tongue can be damaged as sequelae of infection, surgical procedures, or head trauma [88,89]. A direct manifestation of nerve injury, irrespective of etiology, is a disturbance in gustatory function, with hypogeusia and metallic taste commonly reported [90,91,92,93]. In addition, patients with nerve damage experience somatosensory alterations [94,95,96,97]. Although taste dysfunction can be transient, it can persist in some cases of nerve injury [91] and only recover in the long term [98].

Studies investigating biological consequences of nerve injury point toward potential roles of Hh signaling in nerve maintenance. Electron microscopic examination of the CT nerve, after a middle ear infection, revealed alterations in Schwann cells and in integrity of the nerve fibers, per se [99]. We have shown that Schwann cells are Hh-responding cells in rodents [9] and hypothesize that Hh signaling via a nerve source of Shh maintains Schwann cells. Thus, damage to the CT nerve as a source of Shh could compromise Hh signaling in nerves and Schwann cell function.

Furthermore, CT damage during middle ear surgery can result in dry mouth syndrome, due to inadequate saliva secretion [100] and decreased gustatory sensitivity [101]. However, the parotid glands are reportedly not altered. Since parotid saliva contains Shh and has been implicated in TB growth and development [102] there may be a contribution to TB/nerve regeneration. In fact, the presence of Shh in human saliva could function in maintaining normal homeostasis. Additionally, reports of Shh in nasal mucus and reduced levels in hyposmic patients suggest possible Hh pathway functions in olfaction [103].

In surgical patients the TB recover once the CT nerve regenerates after surgery [93,104], followed by recovery of taste sensation [105,106,107]. Neurotrophins, particularly BDNF, have been reported to mediate the nerve regeneration after CT section in mice [57,58]. Shh can activate BDNF in several in vivo and in vitro systems [60,61,62] and, thus, could promote nerve regeneration. We have shown that after CT/LN nerve cut in mice, the FP retain TB remnants and associated Shh [69,70]. In addition, Shh acts as a macrophage chemoattractant in the gut [108] and the newly recruited macrophages can clear myelin debris for axon regeneration [109]. Thus, there are several avenues for Shh signaling in TB and nerve regeneration.

Damage to the GL or CT nerve can also induce phantom sensations, which occur without stimulation [88] and are attributed to disinhibition of remaining gustatory fibers after nerve damage and increased activity in brainstem neurons [94]. In HPI in rodents, nerve responses to chemicals/tastants are eliminated, whereas responses to lingual touch and cold remain [5,6,7]. This presumably leads to a radically altered oral sensory world not only in rodents but also in patients who use HPI drugs [85,86]. Central effects of peripheral taste organ disruption in these and other patients are expected. With Hh signaling inhibition in patients who use Hh pathway antagonist drugs [85,86], peripheral taste sensation is disrupted with possible perceptual dominance of tactile and temperature sensation. Other circumstances of peripheral taste organ and nerve disruption are numerous [88,110] and we suggest that, in many of these disturbances, Shh signaling has potential roles to facilitate regeneration and, thus, taste recovery.

## 9. Concluding Remarks: Hh Signaling in Taste Papillae

### 9.1. Shh and Signaling in Gustatory Papillae, the TBs and in Nerves: Necessary and Sufficient

The Hh pathway is active in regulating peripheral taste organs, the gustatory papillae, taste buds, and neural responses/sensory function. There is ample evidence that Hh signaling is essential for maintaining TB, FP morphology, and taste sensation, but not lingual touch or cold sensation (Figure 2 and Figure 3). On the other hand, it is clear that taste innervation is necessary for TB maintenance and regeneration and a specific role for Shh-positive nerve fibers in sustaining TB has been presented [3,4]. We propose that an intact lingual epithelium and an intact innervation each is necessary but only both together are sufficient for TB homeostasis and sensation (Figure 4) and their functions are not redundant.

### 9.2. Roles for the Basal Lamina

For signaling from Shh in TB and/or in nerves to Hh-responding target cells throughout the taste organ, the nature of the Shh ligand (able to diffuse and signal over long distances) is salient (Figure 4). Signaling over distances that encompass dimensions of the FP would be readily tractable [1,18]. However, if Shh from TB cells is to signal beyond the epithelium to *Gli1^lacZ^* fibroblasts, the molecule would need to (a) be sequestered within or cross the basal lamina or (b) signal via lamellipodia protruding into the basal lamina and, thus, to stromal cells with basal lamina proximity (Figure 5). Similarly, to access Hh-responding Schwann cells, unless these remain on nerves that are within the epithelium, Shh diffusing beyond the basal lamina would be required. On the other hand, Shh within ganglion neurons or nerve fibers could readily access *Gli1^lacZ^*+ satellite, Schwann, or stromal cells, but to signal to FP basal epithelial or perigemmal cells would necessitate sequestration or crossing the basal lamina (Figure 4). The extent to which the basal lamina is a barrier or permissive to Shh in TB homeostasis is not clear (see Section 7). We propose that in the joint signaling from ligand in TB and nerves, that is necessary and sufficient for TB homeostasis, the basal lamina is sequestering Shh and/or permissive to ligand diffusion.

### 9.3. Proposed Roles for Neural Shh in Peripheral Taste Organ Function

With the recent demonstrations of Shh ligand in GG neurons and CT nerve fibers, and in TG soma and oral nerve fibers [3,4,6,75], the taste field is alerted to considering specific roles for neural Shh in peripheral oral functions in addition to TB maintenance. There are several options based on recent data [6,9], as follows: (i) Within the GG, Shh in ganglion neurons can signal to *Gli1^lacZ^*+ satellite cells and sustain overall ganglion integrity. (ii) Within the tongue and the FP core there is potential signaling from the Shh ligand to Hh-responding *Gli1^lac^+* Schwann cells that surround nerve bundles. This pathway activity could participate in maintaining lingual nerves and promoting fasciculation, Schwann cell proliferation, and regeneration and repair. (iii) In the FP stromal core, the Shh in nerves can signal to Hh-responding *Gli1^lacZ^*+ fibroblast cells to participate in supporting connective tissue cells, stimulating production of matrix molecules, and maintaining FP morphological integrity. (iv) Considering LN only, and signaling to Hh-responding *Gli1^lacZ^+* basal epithelial cells of the FP, activities in these target cells would logically function to maintain papilla structure. (v) Furthermore, a diversity of nervous system processes regulated by Shh have been reviewed [111] and these suggest functions for Shh in axon guidance and as a chemoattractant for TB innervation. Within the TB, the local Shh might interact with Schwann cells wrapping the nerves and affect aspects of synapse plasticity, however, very few nerve fibers within the TB are myelinated [112], so a prevalence of Schwann cells might not be expected. All of these varied and proposed roles could involve canonical or noncanonical signaling, but many more studies will be required to address the proposals.

### 9.4. Summary and Future Directions

Building from studies of embryonic development that led to versatile postnatal experimental approaches, it is firmly established that there are essential roles for the Hh pathway in maintenance and regeneration of taste organs and taste and oral sensation. However, there is much that remains unknown when confronting the breadth of responses to such a prominent pathway in gustatory cell tissue and functional maintenance. Unique characteristics in the FP/TB and CT nerve electrophysiological responses, after Hh signaling disruption, disclose a particular multimodal nature of the FP and CT-innervated sensory organs, and in regulation of oral sensation. The gustatory and somatosensory function of the CT and innervated oral epithelia are of special salience to patients treated with Hh pathway inhibiting drugs and functional sensory effects should be investigated. Important also in future investigations are more thorough explorations of the many molecules in the Hh pathway and consideration of canonical and noncanonical signaling. Since the Hh ligand is located in TB, and in nerves and neurons that innervate taste papillae and TB, a focus on signaling within specific compartments and niches in gustatory organs will be fruitful. New roles for Hh/nerve interactions should receive attention, in addition to signaling within the connective tissue elements of taste organs. Analysis of proposed functions for Hh signaling in taste/lingual innervation could reveal how the Hh pathway attracts, supports, and guides nerves of the tongue in oral sensation. The taste organ is a heterogeneous and complex array of dynamic cells and tissues that include epithelia, sensory cells, nerve fibers and neurons, and stromal cells and extracellular matrix components. These all are potentially regulated by Hh signaling in diverse mechanisms.

## Abbreviations

FP	Fungiform papilla
CV	Circumvallate papilla
TB	Taste bud
CT	Chorda tympani
GL	Glossopharyngeal nerve
LN	Lingual nerve
Shh	Sonic hedgehog
Hh	Hedgehog
GG	Geniculate ganglion
TG	Trigeminal ganglion
